# Regulation and Regulatory Role of WNT Signaling in Potentiating FSH Action during Bovine Dominant Follicle Selection

**DOI:** 10.1371/journal.pone.0100201

**Published:** 2014-06-17

**Authors:** P. S. P. Gupta, Joseph K. Folger, Sandeep K. Rajput, Lihua Lv, Jianbo Yao, James J. Ireland, George W. Smith

**Affiliations:** 1 Laboratory of Mammalian Reproductive Biology and Genomics, Michigan State University, East Lansing, Michigan, United States of America; 2 Department of Animal Science, Michigan State University, East Lansing, Michigan, United States of America; 3 College of Animal Science and Technology, Shanxi Agricultural University, Taigu, Shanxi, China; 4 Laboratory of Animal Biotechnology and Genomics, Division of Animal and Nutritional Sciences, West Virginia University, Morgantown, West Virginia, United States of America; University of Missouri, United States of America

## Abstract

Follicular development occurs in wave like patterns in monotocous species such as cattle and humans and is regulated by a complex interaction of gonadotropins with local intrafollicular regulatory molecules. To further elucidate potential mechanisms controlling dominant follicle selection, granulosa cell RNA harvested from F1 (largest) and F2 (second largest) follicles isolated at predeviation (PD) and onset of diameter deviation (OD) stages of the first follicular wave was subjected to preliminary RNA transcriptome analysis. Expression of numerous WNT system components was observed. Hence experiments were performed to test the hypothesis that WNT signaling modulates FSH action on granulosa cells during follicular waves. Abundance of mRNA for WNT pathway members was evaluated in granulosa cells harvested from follicles at emergence (EM), PD, OD and early dominance (ED) stages of the first follicular wave. In F1 follicles, abundance of *CTNNB1* and *DVL1* mRNAs was higher and *AXIN2* mRNA was lower at ED versus EM stages and *DVL1* and FZD6 mRNAs were higher and *AXIN2* mRNA was lower in F1 versus F2 follicle at the ED stage. Bovine granulosa cells were treated in vitro with increasing doses of the WNT inhibitor IWR-1+/− maximal stimulatory dose of FSH. IWR-1 treatment blocked the FSH-induced increase in granulosa cell numbers and reduced the FSH-induced increase in estradiol. Granulosa cells were also cultured in the presence or absence of FSH +/− IWR-1 and hormonal regulation of mRNA for WNT pathway members and known FSH targets determined. FSH treatment increased *CYP19A1*, *CCND2*, *CTNNB1*, *AXIN2* and *FZD6* mRNAs and the stimulatory effect on *CYP19A1* mRNA was reduced by IWR-1. In contrast, FSH reduced *CARTPT* mRNA and IWR-1 partially reversed the inhibitory effect of FSH. Results support temporal and hormonal regulation and a potential role for WNT signaling in potentiating FSH action during dominant follicle selection.

## Introduction

The precise mechanisms regulating the species-specific number of follicles that grow and ovulate while all other growing follicles undergo atresia are poorly understood. In single-ovulating species like cattle and humans, antral follicle growth occurs in a characteristic wave-like pattern [1]–[3]. The onset of each follicular wave is preceded by a transient increase in serum FSH that promotes emergence of a cohort of small antral follicles. In the face of declining FSH concentrations, typically, a single dominant follicle out of this cohort continues to grow to ovulatory size and produces increased amounts of estradiol. The remaining smaller “subordinate” follicles lose the capacity to produce estradiol and die via atresia. Selection of a single dominant follicle is an evolutionarily conserved mechanism critical to control of number of offspring per pregnancy in monotocous species.

While the key role of pituitary gonadotropins in mediating the wave like pattern of follicular development is well established, the intrafollicular mechanisms and regulatory molecules that are obligatory for selection of a single dominant follicle during each follicular wave have not been fully established. Enhanced capacity of the dominant follicle to produce estradiol is critical to sustain dominant follicle growth and initiate estrous behavior, the preovulatory gonadotropin surge, resumption of meiosis and ovulation [4]. Estradiol production during follicular development in cattle is regulated by diverse hormones and growth factors including FSH [3], IGF1 [3], [5], [6], CART [7], BMP2 [8] as well as estradiol itself [9]. Recently it has been shown in rodents that estradiol production is regulated by members of the wingless-type mouse mammary tumor virus integration site (WNT) signaling family which modulate FSH action [10], [11]. Although WNT is essential for ovarian development [12], its mechanistic role in regulation of folliculogenesis in the adult ovary, particularly in the context of follicular waves, is not well understood.

WNTs are secreted ligands that transduce their signals by binding to the frizzled (FZD) family of G protein-coupled receptors (reviewed in [12]). The WNTs control multiple developmental processes including cell fate specification, proliferation, differentiation and apoptosis, and WNT dysregulation has been implicated in the development of cancers, particularly colorectal cancers [13]. The members of the WNT family act through three different pathways; the canonical WNT/β-catenin (CTNNB1) pathway, the non-canonical planar cell polarity pathway and the WNT/Ca2+ pathway. In an unstimulated cell, AXIN2 will bind to CTNNB1 in a complex with GSK3β and APC which leads to rapid proteolytic degradation of CTNNB1. In the canonical pathway, a WNT ligand will bind to its FZD receptor and to an LDL-receptor-related protein (LRP) co-receptor, which will activate Disheveled 1 (DVL1) to bind to AXIN2 and disrupt the formation of the AXIN2/GSK3β/APC/CTNNB1 complex. This stops the proteolytic degradation of CTNNB1, so CTNNB1 can enter the nucleus and initiate transcription of regulated genes [12].

In the current studies, a preliminary RNA transcriptome sequencing study designed to further elucidate potential mechanisms controlling dominant follicle selection revealed expression of multiple WNT ligands, receptors and cognate intracellular signaling molecules in bovine follicles harvested at specific stages of a follicular wave. Based on these results, we hypothesized that WNT signaling is a key mediator of FSH action linked to dominant follicle selection in cattle. To begin to test this hypothesis, the temporal and hormonal regulation of mRNA for specific WNT and FZD molecules and associated pathway components in bovine granulosa cells were tested and the effects of a small molecule inhibitor of WNT signaling on FSH action in bovine granulosa cells were investigated. Results support a potential role for WNT signaling in regulation of FSH action and ovarian follicular selection during follicular waves in cattle.

## Materials and Methods

### Ethics Statement

All animal procedures were performed with approval of the Michigan State University IACUC.

### Follicle Collection at Specific Stages of Follicular Wave

Non lactating Holstein dairy cows (n = 18) were given two injections of prostaglandin F2α 14 days apart to synchronize their estrous cycles and randomly assigned to a time point for ovariectomy. Beginning 2 days after the second prostaglandin F2α injection, ovaries were scanned 2–3 times daily using ultrasonography to monitor ovulation and initiation and growth of the first wave of follicles. Follicles were collected at specific stages of the follicular wave (including at emergence [EM; first scan where a follicle ≥4 mm is detected; usually the same day as ovulation; n = 3], pre-deviation [PD; 1.5 d after EM; ∼day 3 of the estrous cycle; n = 5], onset of diameter deviation [OD; first scan where growth of the F1 (largest; future dominant) follicle to >8.5 mm was detected and the F2 (second largest; future subordinate) follicle was still growing; ∼day 4 of the cycle; n = 5], and early dominance stage [ED; first scan where one follicle in the cohort was 2 mm larger than others; ∼day 5 of the cycle; n = 5]) as described previously [9]. Granulosa cells were isolated from the F1 and F2 follicles and immediately lysed and stored at −80°C.

### RNA Isolation

Total RNA was isolated from the lysates using the RNeasy mini kit (Qiagen) and DNase treated on column following the manufacturer’s protocol. Total RNA (100 ng/sample) was then converted into cDNA using the iScript cDNA synthesis kit (Bio-Rad) following the manufacturer’s instructions. Nuclease free water was then added to dilute the cDNA to a total of 40 µl.

### Q-PCR

Quantitative PCR was performed using duplicate 12.5 µl reactions containing 6.25 µl of SsoAdvanced SYBR Green supermix (Biorad), 300 nM of forward and reverse primer (0.75 µl each), 1 µl of cDNA and 3.75 µl of nuclease free water. Primers were designed using either PerlPrimer or Primer express and reactions were run on a CFX96 touch instrument (Bio-Rad) for 40 cycles of 95°C for 15 s followed by 60°C for 1 min. Relative expression values (ΔΔCT) were calculated using CFX manager 3.0 software (Bio-Rad), using RPS18 as the housekeeping gene. Primer sequences can be found in Table 1.

**Table 1:** Sequence of primers for real time RT-PCR.

Gene	Genebank Accession number	Primer sequence
AXIN2	NM_001192299	F: 5′- AGCGGATACAGGTCCTTCAG -3′
		R: 5′- GTCACTGGATATCTCGCTGTC -3′
CTNNB1	NM_001076141	F: 5′- AAGGTGTGGCAACATATGCAG -3′
		R: 5′- GGGCACCAATATCAAGTCCA -3′
CCND2	NM_001076372	F: 5′- TGACTTCAAGTTTGCCATGTACC -3′
		R: 5′- TTTGAGGCAATCCACATCGG -3′
DVL1	NM_001206601	F: 5′- CACTCAACATGGAGAGGCAC -3′
		R: 5′- GAAGTTGACGTCGTTCACCT -3′
FZD6	XM_003586904	F: 5′- CATGTCCTTATCAGGCAAATACAG -3′
		R: 5′- TTGCTTCCAACCCAGAAGAC -3′
CYP19A1	NM_174305	F: 5′- CACCCATCTTTGCCAGGTA -3′
		R: 5′- ACCCACAGGAGGTAAGCCTAT -3′
CARTPT	NM_001007820	F: 5′- TGTGACTGTCCCCGAGGAA -3′
		R: 5′- GAAGCGTGGGTGCCTCATA -3′
ACTIN	BG689033	F: 5′- GGATGAGGCTCAGAGCAAGAGA -3′
		R: 5′- TCGTCCCAGTTGGTGACGAT -3′
RPS18	NM_001033614	F-5′- GTGGTGTTGAGGAAAGCAGACA-3′
		R-5′- TGATCACACGTTCCACCTCATC-3′

### RNA Sequencing

RNA sequencing was performed by the W.M. Keck Center for Comparative and Functional Genomics at the University of Illinois at Urbana-Champaign. RNA samples from four F1 and F2 follicles at each stage (PD and OD; 4 animals per group) were pooled within each stage and size classification and RNA-Seq libraries were prepared with a TruSeq RNA Sample Preparation kit (Illumina) according to the manufacturer’s instructions. The pooled samples were sequenced on two lanes for 100 cycles on a HiSeq2000 using a TruSeq SBS kit V5 (Illumina) and analyzed with pipeline version 1.8. The CLC Genomics Workbench (CLC bio, Aarhus, Denmark) was used to map the sequence reads to the bovine RefSeq database. RPKM values were calculated for expression of genes in follicles among different stages.

### Granulosa Cell Culture

Three experiments were performed using our previously described [14] granulosa cell culture system where cells respond to FSH with a dose-dependent increase in estradiol and display increased estradiol production with time in culture [15]. Briefly, granulosa cells were isolated from ovaries of abattoir origin (JBS Packerland, Plainwell, MI) and cultured for 6 days in media (MEMα; Invitrogen, Carlsbad, CA) containing insulin (1 ng/ml), transferrin (5 µg/ml), IGF1 (2 ng/ml), sodium selenite (4 ng/ml), androstenedione (10^−6^ M; all from Sigma, St. Louis, MO), penicillin (100 IU/ml), streptomycin (0.1 mg/ml) and fungizone (0.625 µl/ml; Invitrogen). For all experiments, granulosa cells (100,000 live cells per well) were plated in 96 well culture dishes (BD Biosciences, San Jose, CA). The first experiment examined the effect of the WNT signaling inhibitor IWR-1 on basal and FSH-induced estradiol production and cell numbers. The WNT inhibitor stabilizes the interaction of AXIN2 with CTNNB1 leading to degradation of CTNNB1 and inhibition of the canonical WNT pathway [16]. Treatments consisted of culture medium with DMSO (diluent control group) or medium containing 0.1, 1.0 or 10 µM of IWR-1 (Cayman Chemical) with or without the addition of maximal stimulatory dose of FSH (0.5 ng/ml; NHPP) for 6 days with 12 wells per treatment in each replicate experiment. Media was changed every 2 days. On the 6^th^ day of culture, media was removed and stored at −20°C until analysis for estradiol concentrations and the cells were washed, trypsinized and counted using a Coulter counter (Beckman Coulter) set to count cells between 5 and 20 µm in size, as previously described [15]. The experiment was replicated 4 times using ovaries obtained on different days.

In the second experiment, the effect of FSH and maximal inhibitory dose of IWR-1 on mRNA abundance for select WNT pathway members and other modulators of FSH action were determined. In this experiment, granulosa cells were treated (24 wells per treatment) with DMSO (vehicle control) or the maximally effective dose of IWR-1 (1 µM) in the presence or absence of FSH (0.5 ng/ml). On the 6^th^ day of culture, media was removed and stored at −20°C until analysis for estradiol concentrations and the cells were lysed and preserved at −80°C until processed for total RNA isolation. The experiment was replicated 4 times using ovaries obtained on different days.

In the third experiment, the effect of IWR-1 treatment on CTNB1 and AXIN2 protein abundance was determined. For this experiment, granulosa cells were isolated and cultured as described for experiment 2. On the 6^th^ day of culture, media was removed and stored at −20°C until analysis for estradiol concentrations. The cells were then washed, aspirated from the wells and centrifuged at 3000 g for 5 min. The cell pellet was then snap frozen in liquid nitrogen and preserved at −80°C until Western blot analysis.

### Radioimmunoassay

Concentrations of estradiol [15] in media samples were estimated using commercially available Coat-a-Count kits (Siemens Medical Diagnostics, Los Angeles CA). Intra- and inter- assay coefficients of variation were 7.8% and 9.1%, respectively.

### Western Blot Analysis

Cell pellets were thawed on ice in RIPA buffer (Sigma) containing 1X protease inhibitor cocktail (Roche Applied Science) for 30 min at 4°C. Lysates were subjected to high intensity sonication (3 sets of 30 second pulses) and the insoluble materials were removed by centrifugation at 15,000 rpm for 15 min at 4°C. The total protein concentration for supernatants was determined using a DC protein assay kit (Bio-Rad). Ten micrograms of protein extract per sample was resolved under reducing conditions on 4–20% TGX gels (Bio-Rad) by SDS-PAGE electrophoresis and transferred to polyvinylidene fluoride membranes (Millipore). After blocking with Pierce protein-free blocking buffer (Thermo Scientific) at room temperature (RT) for 1 h, the membranes were incubated overnight at 4°C in protein free blocking buffer with appropriate primary antibodies and subsequently incubated with horseradish peroxidase (HRP)-conjugated secondary antibodies. SuperSignal west Dura Chemiluminescent substrate (Thermo Scientific) was used for visualization, and signal was captured on Hybond-CL autoradiography film (Denville Scientific). The band intensities for CTNNB1 and AXIN2 were determined using Image J software and normalized relative to corresponding total-actin level in each lane. Membranes were probed sequentially with primary rabbit anti-Axin2 polyclonal antibody [1∶1000 (vol/vol), Sigma-Aldrich; #SAB3500619] and rabbit anti-β-catenin polyclonal antibody [1∶1000 (vol/vol), Cell Signaling Technology; #9581]. After detection of AXIN2 and CTNNB1, membranes were striped with restore Western blot stripping buffer (Thermo Scientific) for 30 min at RT and reprobed with mouse anti-actin monoclonal antibody (1∶5000 (vol/vol) Millipore; #MAB1501). HRP-conjugated Ant-rabbit-IgG (Cell Signaling Technology] and Anti-mouse-IgG (Thermo Scientific) were used as secondary antibodies at a 1∶5000 (vol/vol) dilution.

### Statistical Analysis

All data except for the RNA sequencing experiment (described above) were analyzed in a one way ANOVA using Proc GLM in SAS version 9.2 [17]. Data were log transformed before analysis if required for assumptions of normality and differences between means were determined using Fisher’s Protected LSD. Data are presented as untransformed mean ±SEM.

## Results

### WNT Signaling Pathway is Involved in Selection of the Dominant Follicle

**Figure 1:**
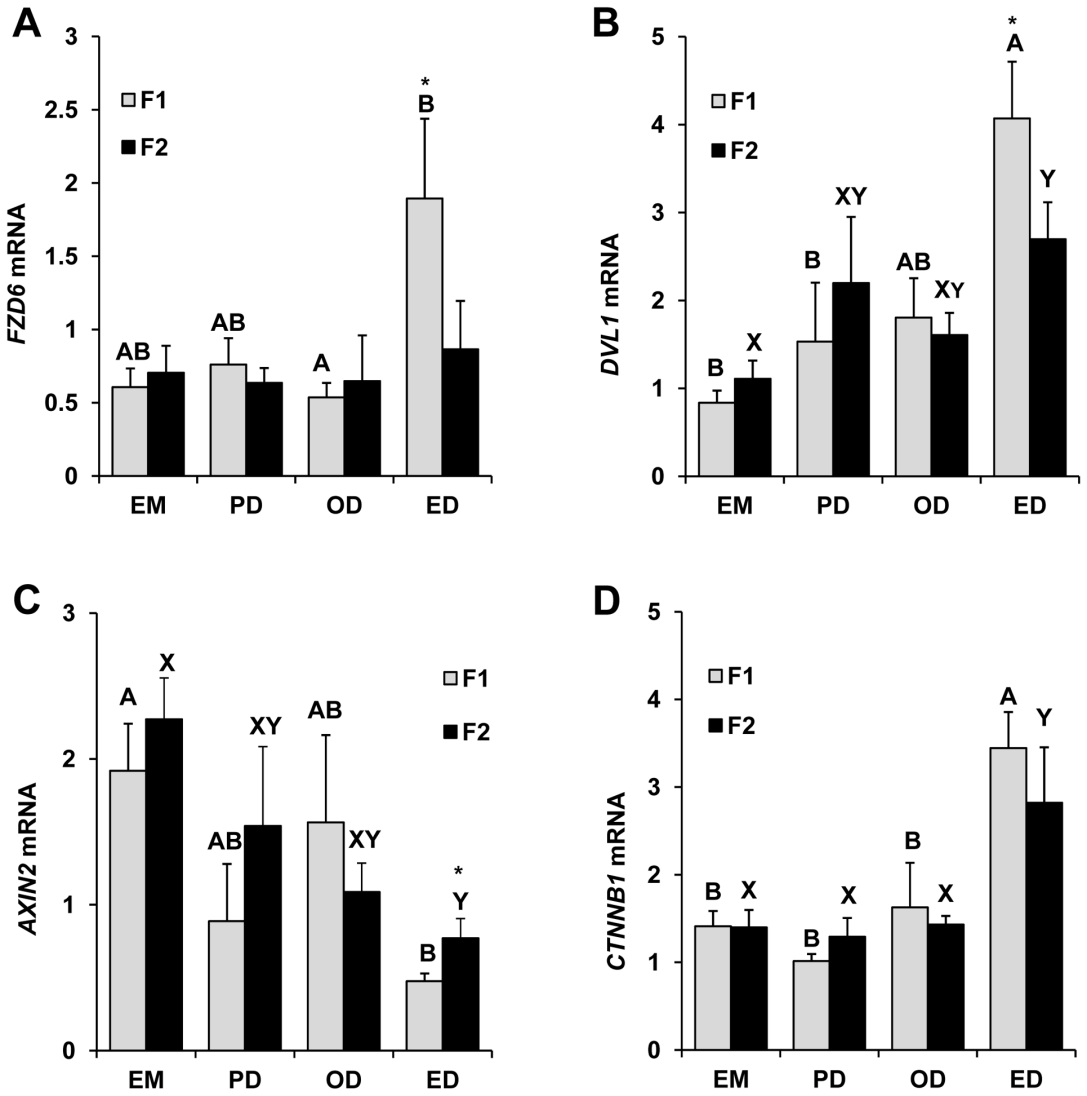
Temporal expression profile of select WNT system components in granulosa cells harvested from F1 and F2 follicles at the emergence (EM; n = 3 animals), predeviation (PD; n = 5 animals), onset of deviation (OD; n = 5 animals) and early dominance (ED; n = 5 animals) stages of the first follicular wave Relative mRNA abundance for *FZD6* (A), *DVL1* (B), *AXIN2* (C) and *CTNNB1 (D)* was determined by Q-RTPCR. RNA expression data was normalized relative to *ACTIN* as internal control. ^A,B^ P<0.05 for transcript abundance in F1 follicles across stages of follicular wave; ^X,Y^ P<0.05 for transcript abundance in F2 follicles across stages of follicular wave; *P<0.05 for transcript abundance between F1 versus F2 follicles within stage of follicular wave.

Preliminary RNA sequencing based comparative transcriptome analysis of bovine follicles at predeviation (PD) and onset deviation (OD) stages has demonstrated expression of numerous WNT signaling pathways members including WNT ligand molecules, frizzled receptors and catenins (Table S1). Based on this observation, temporal expression of select members (*CTNNB1, AXIN2, DVL1* and *FZD6*) of interest of the WNT system was further examined by real-time PCR using total RNA isolated from largest (F1) and second largest (F2) follicles collected at four different stages of a follicular wave. In the F1 follicles, relative abundance of *FZD6* mRNA was ∼2 fold greater (P<0.05) in follicles collected at ED stage relative to OD stage (Figure 1A). Abundance of mRNAs for *DVL1* (Figure 1B) and *CTNNB1* (Figure 1D) was significantly greater (P<0.05) and that of *AXIN2* (Figure 1C) was ∼2 fold lower (P<0.05) during the ED stage compared to EM stage. Real time PCR analysis of F2 follicles revealed similar expression pattern for *DVL1, AXIN2* and *CTNNB1* (Figure 1B-D) transcripts as observed in F1 follicles, however *FZD6* transcript showed no significant variation in expression among different stages of follicular development in F2 (Figure 1A). Furthermore, mRNA abundance for *FZD6* and *DVL1* was higher (P<0.05) and *AXIN2* was lower (P<0.05) in the F1 (dominant) versus F2 (subordinate) follicles at the early dominance stage (immediately after dominant follicle selection; Figure 1A and 1C).

### Functional Role of WNT Signaling Pathway in Potentiating FSH Action during Dominant Follicle Selection

**Figure 2:**
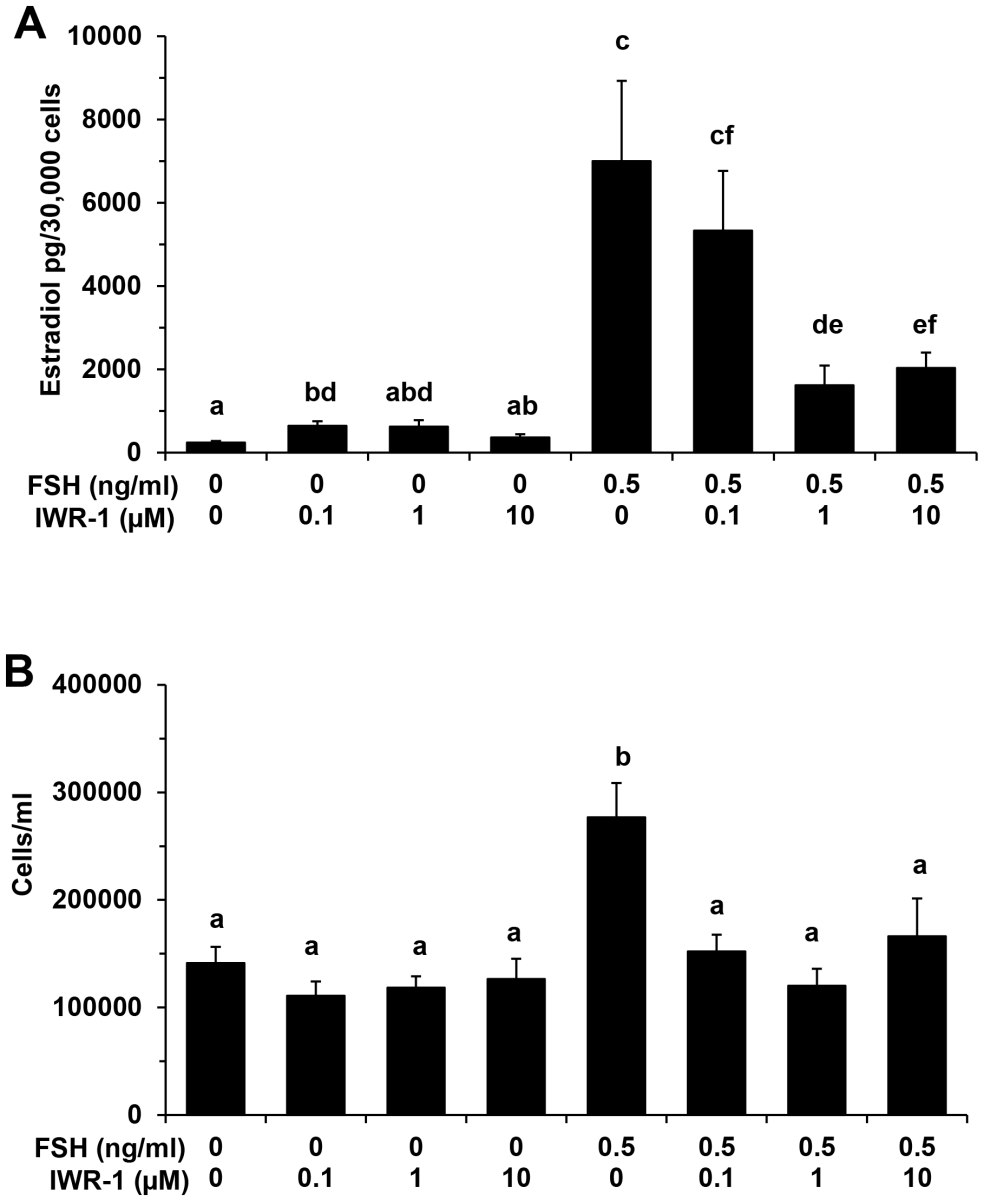
Effect of increasing concentrations of WNT signaling inhibitor IWR-1 on FSH stimulated estradiol production (A) and cell numbers (B) for bovine granulosa cells. Granulosa cells were treated with 0, 0.1 µM, 1.0 µM and 10 µM concentration of IWR-1 in the presence and absence of maximal stimulatory dose (0.5 ng/ml) of FSH. Each column represents mean± SEM for four independent experiments. ^a,b,c,d,e,f^ P<0.05.

Above results support distinct temporal regulation of WNT system members during dominant follicle selection and are suggestive of a regulatory role. Given the prominent role for FSH in regulation of follicular waves in cattle, the potential contribution of WNT signaling to FSH action on bovine granulosa cells was examined. When cells were treated with increasing concentrations of the WNT signaling inhibitor IWR-1 in the absence of FSH, a slight increase in estradiol production was observed only at the 0.1 µM dose (P<0.05; Figure 2A). However, IWR-1 treatment in the presence of maximal stimulatory dose of FSH (0.5 ng/ml) resulted in a dose dependent inhibition of the stimulatory effect of FSH with >75% inhibition (Figure 2A) observed at the maximal inhibitory dose of IWR-1(1 µM; P<0.05). In contrast, no effect of IWR-1 treatment on numbers of granulosa cells obtained at the end of culture (in the absence of FSH) was observed, whereas the FSH-induced increase in granulosa cell numbers was blocked in response to all doses of IWR-1 tested (Figure 2B; P<0.05).

**Figure 3:**
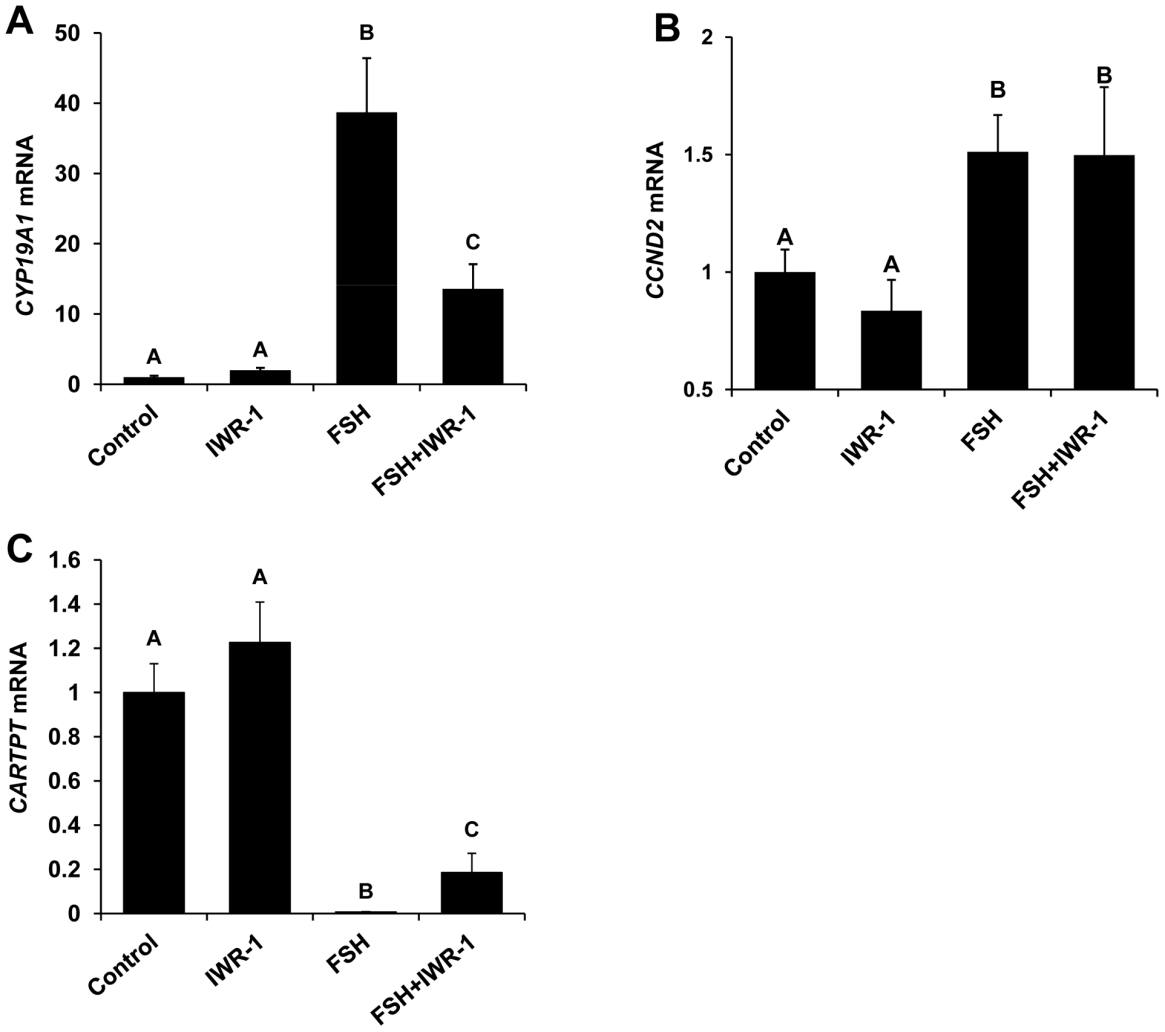
Effect of IWR-1 mediated WNT signaling inhibition on FSH-induced regulation of FSH target gene (*CYP19A1*, *CCND2* and *CARTP*T) mRNA expression in bovine granulosa cells. Quantitative RT-PCR analysis was performed to determine the effects of treatment with 0 or 0.5 ng/ml FSH in the presence or absence of 1.0 µM IWR-1 on *CYP19A1 (A)*, *CCND2 (B) and CARTPT (C)* mRNA expression in cultured granulosa cells. Expression of mRNA for genes of interest was normalized relative to abundance of mRNA for *ACTIN* as internal control. Bars represent mean +/− SEM for four experiments. ^A,B^ P<0.05.

To further elucidate the role of WNT signaling in potentiating FSH action, the effect of IWR-1 treatment on FSH-induced regulation of the known FSH target genes *CYP19A1*, *CCND2* and *CARTPT* was investigated using bovine granulosa cells cultured in the presence or absence of FSH and maximal inhibitory dose of IWR-1. Effects of IWR-1 treatment on mRNA abundance for *CYP19A1*, *CCND2* and *CARTPT* were not observed for cells cultured in the absence of FSH (Figure 3). Treatment with FSH increased mRNA abundance for *CYP19A1* and *CCND2.* The stimulatory effects of FSH on *CYP19A1* (Figure 3A) were partially reversed in response to WNT signaling inhibitor (IWR-1) treatment (P<0.05), but IWR-1 treatment did not inhibit FSH stimulation of *CCND2* mRNA (Figure 3B). In contrast, IWR-1 treatment partially reversed the FSH-induced inhibition of granulosa cell *CARTPT* mRNA expression (Figure 3C; P<0.05).

**Figure 4:**
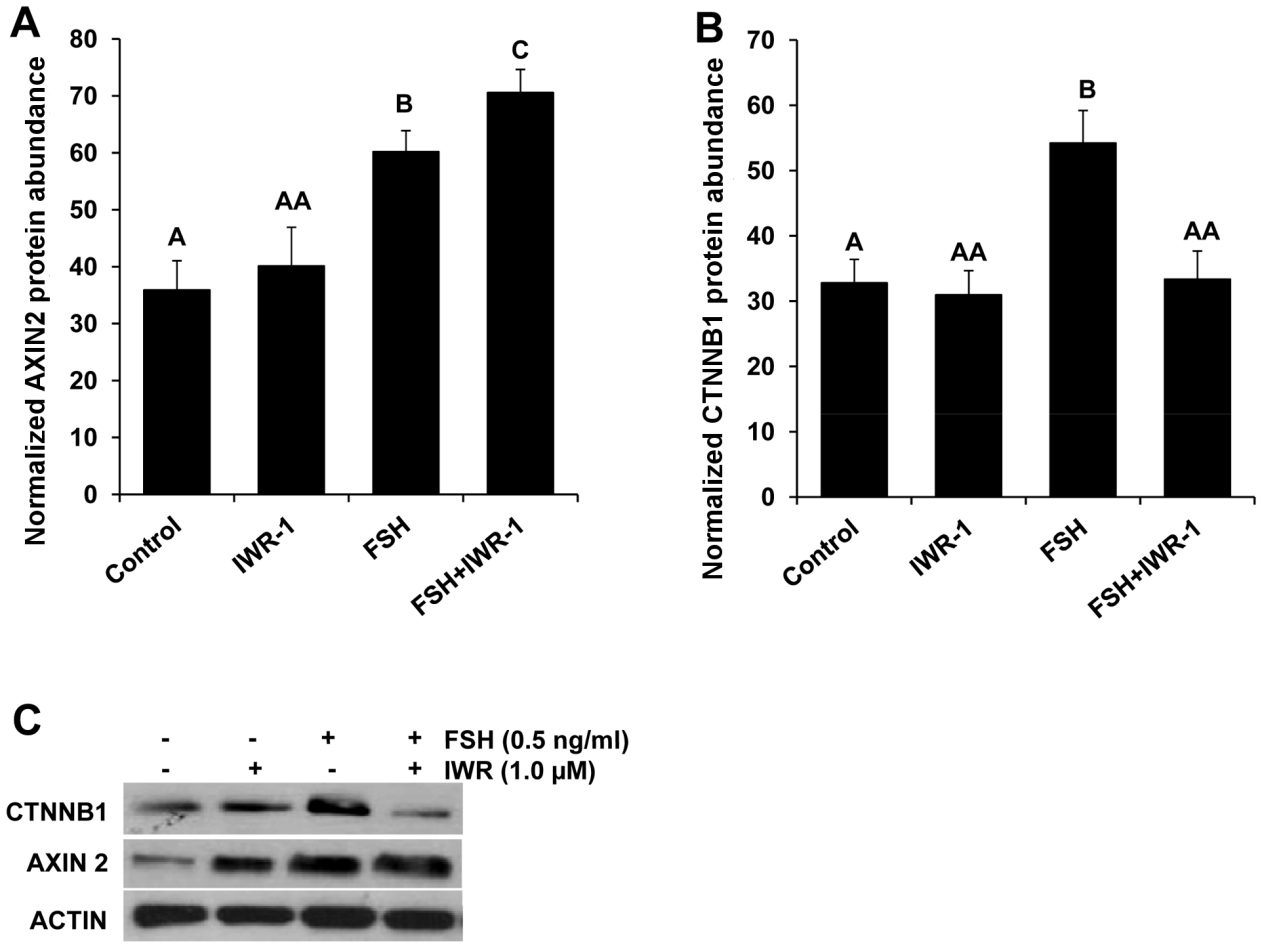
Effect of FSH and WNT signaling inhibitor (IWR-1) treatment on CTNNB1 and AXIN2 protein abundance in bovine granulosa cells. Relative abundance of AXIN2 (A) and CTNNB1 (B) proteins (as determined by Western blot analysis and densitometry) in bovine granulosa cells cultured in the presence or absence of 0.5 ng/ml FSH plus 1.0 µM IWR-1. Results depict mean +/− SEM for four replicate experiments with relative protein abundance for CTNNB1 and AXIN2 normalized relative to ACTIN. ^A,B,C^ P<0.05.

To confirm IWR-1 mechanism of action in inhibition of WNT signaling in bovine granulosa cells, Western blot analysis for CTNNB1 and AXIN2 was conducted. Results revealed a significant increase in AXIN2 in granulosa cells treated with FSH plus IWR-1 relative to cells treated with FSH alone (Figure 4A). Furthermore, an approximately 1.8 fold (P<0.05) increase in CTNNB1 protein was observed in granulosa cells treated with FSH compared to untreated controls. The FSH-induced increase in CTNNB1 protein was completely blocked in response to IWR-1 treatment (Figure 4B) supporting IWR-1 effects on CTNNB1 protein accumulation.

### Hormonal Regulation of WNT Signaling Pathway Members in Bovine Granulosa Cells

**Figure 5:**
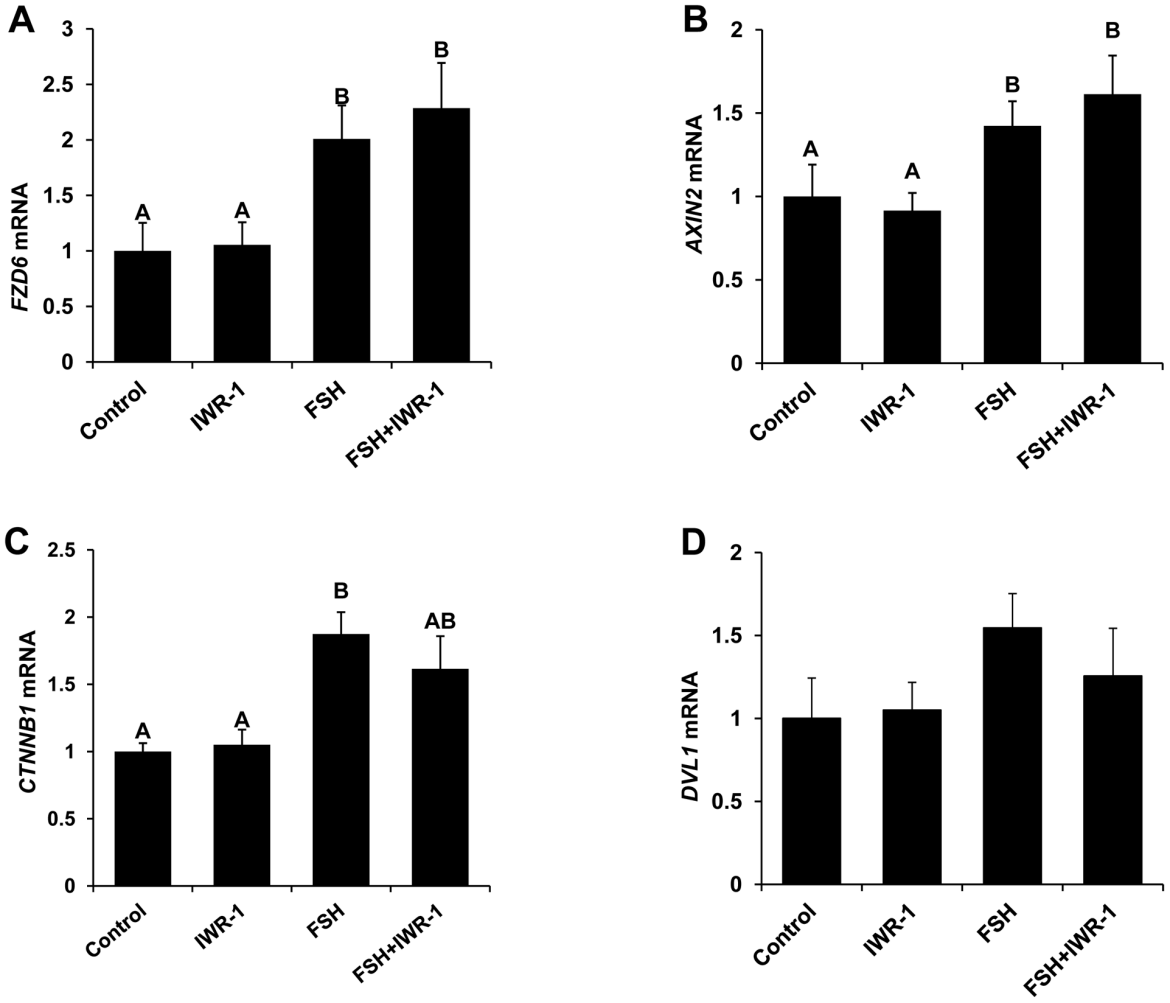
Effect of FSH and WNT signaling inhibitor (IWR-1) treatment on *FZD6*, *AXIN2*, *CTNNB1* and DVL1 *mRNA* abundance in bovine granulosa cells. Quantitative RT-PCR analysis was performed to determine the effects of treatment with 0 or 0.5 ng/ml FSH in the presence or absence of 1.0 µM IWR-1 on *FZD6 (A)*, *CCND2 (B) and CARTPT (C)* mRNA expression in cultured granulosa cells. Expression of mRNA for genes of interest was normalized relative to abundance of mRNA for *ACTIN* as internal control. Bars represent mean +/− SEM for four experiments. ^A,B^ P<0.05.

Above results support a prominent role for WNT signaling in potentiating specific components of FSH action in bovine granulosa cells. To further understand the potential functional role in follicular selection, the hormonal regulation of expression of select WNT system components in bovine granulosa cells was investigated. Granulosa cells cultured in the presence of FSH exhibited increased expression of *AXIN2*, *CTNNB1* and *FZD6* mRNA (Figure 5A-C) compared to untreated controls (P<0.05), but no effect of inhibition of WNT signaling via IWR-1 treatment on basal and FSH-induced mRNA expression for *AXIN2*, *CTNNB1* and *FZD6* was observed. In contrast mRNA abundance for *DVL1* was not impacted by FSH or IWR-1 treatment (Figure 5D). Results support WNT-CTNNB1 independent FSH-induced regulation of expression of specific WNT system components in bovine granulosa cells.

## Discussion

In the present study, we identified components of the WNT system expressed in granulosa cells and potentially involved in dominant follicle selection during follicular waves in cattle. We have further established an intriguing regulatory role of WNT signaling pathway in specific aspects of FSH action on bovine granulosa cells associated with follicular growth and steroidogenesis and novel insight on the hormonal regulation of expression of WNT system components in bovine granulosa cells. Collectively, results support additional studies of mechanism of action and functional requirement of WNT signaling for follicular selection in cattle.

Diameter deviation marks the initiation of divergence in growth rate and estradiol producing capacity between the F1 or largest (future dominant) and F2 or second largest (future subordinate) growing follicles resulting in acquisition of follicle dominance [3]. To further elucidate potential regulators of this key developmental transition during follicular waves, RNA transcriptome sequencing was utilized to characterize granulosa cell transcriptome composition in the largest (F1) and second largest (F2) follicles immediately prior to (predeviation; PD stage) versus at onset of diameter deviation (OD stage). Granulosa cell expression of numerous WNT ligands, FZD receptors, catenins and other components of the intracellular signaling pathways was revealed. Comparative transcriptome analysis suggests that WNTs using both the canonical (*WNT2B*, *WNT8B*) and non-canonical (*WNT5A*, *WNT11*, *WNT16*) signaling pathways are present during bovine follicular development.

A transient increase in FSH precedes the onset of each follicular wave and dominant follicle selection occurs in the face of declining FSH concentrations [1]–[3]. RNA transcriptome sequencing results suggest that WNT signaling might play an important role in regulation of follicle growth during follicular waves. Hence, changes in transcript abundance for select canonical pathway members (*FZD6*, *DVL1*, *AXIN2* and *CTNNB1)* at four progressive stages (EM, PD, OD and ED) of follicular development (prior to and after dominant follicle selection) were further investigated. Results demonstrated a significant down regulation of *AXIN2* (essentially required to form CTNNB1 destruction complex) and upregulation of *FZD6*, *DVL1* and *CTNNB1* expression (markers for WNT signaling activation) at ED stage compared to EM stage of follicular development. Furthermore, mRNA for *AXIN2* was lower and for *DVL1* and *FZD6* was higher in the F1 (dominant) versus F2 (subordinate) follicles at the early dominance stage (immediately after dominant follicle selection), further supporting a potential role in dominant follicle selection. Ovarian expression and a prominent role for specific WNT pathway members in ovarian development, folliculogenesis and tumorigenesis in rodents have been established [12]. To our knowledge, expression of WNT pathway components at specific stages of a follicular wave has not been reported previously. Previous studies demonstrated greater abundance of CTNNB1 protein in large bovine follicles of abattoir origin with high versus low follicular fluid estradiol concentrations [18]. Although follicles were not grouped using known criteria indicative of follicle health status [19], [20], size range of follicles analyzed [21] suggest most had reached dominant follicle stage of follicular wave. Collectively, present results support our hypothesis about the potential role of the canonical WNT signaling pathway in regulation of granulosa cell functions associated with dominant follicle selection and (or) dominance.

Pleiotropic actions of FSH are critical to growth of ovarian follicles including regulation of granulosa cell proliferation, estradiol production and mediated by regulation of key target genes [22]. Previous studies support a prominent role for WNT signaling in regulation of ovarian steroidogenesis in rodents [23]. Here, the role of WNT signaling in modulation of multiple components of FSH action on bovine granulosa cells was tested using a chemical inhibitor (IWR-1) that inhibits the canonical WNT pathway by stabilizing the cytoplasmic AXIN2 protein dependent destruction complex and thus inducing the proteosomal degradation of CTNNB1 [24]. Interestingly, we observed a pronounced reduction in cell numbers and estradiol production for FSH treated granulosa cells subjected to WNT inhibitor treatment, which established its functional contribution to FSH action on bovine granulosa cells. A stimulatory effect of FSH on granulosa cell numbers using this culture system has been reported previously [6]. The reduced estradiol production and granulosa cell numbers was accompanied by increased AXIN2 protein levels and inhibition of FSH-induced rise in CTNNB1 protein in FSH treated cells cultured in the presence of IWR-1. However, dramatic effects of IWR-1 treatment in the absence of FSH were not observed. Previous studies using CTNNB1 overexpressing and null mutant mice demonstrated a role for CTNNB1 and WNT signaling in modulation of granulosa cell proliferation and FSH target gene expression in mice [25]. Similarly, a recent study demonstrated that knockdown of CTNNB1, a key member of canonical WNT signaling pathway, in murine granulosa cells compromised the ability of FSH to promote the mobilization of CX43 into gap junctions and consequently reduced gap junction intercellular communication [26].

FSH-induced cell proliferation and estradiol production via granulosa cells are mediated via PKA-induced phosphorylation of CREBP, CTNNB1 and other transcription factors which induce the transcription of *CYP19A1* gene encoding for the aromatase enzyme required to catalyze the conversion of testosterone to estradiol [27], and for *CCND2* and other cell cycle regulators which modulate granulosa cell proliferation [28]. The current and our previous studies [15] demonstrated a dramatic increase in *CYP19A1* transcript abundance in response to FSH treatment and an increase in *CCND2* mRNA was also observed in FSH treated cells. However, inhibition of WNT signaling pathway via IWR-1 treatment significantly reduced the ability of FSH to induce *CYP19A1* mRNA expression leading to a reduction in granulosa cell estradiol production, but did not impact FSH-induced regulation of *CCND2* mRNA. Given this evidence, it is likely that activation of WNT signaling helps mediate FSH stimulated estradiol production in granulosa cells in part by regulating *CYP19A1* transcription through stabilization and nuclear translocation of beta-catenin transcription factor. We also have previously reported a prominent role for *CARTPT* in negative regulation of FSH action in subordinate follicles associated with dominant follicle selection [15], [29]. However, little is known about factors and signaling pathways that regulate *CARTPT* expression. An inhibitory effect of FSH on granulosa cell *CARTPT* mRNA was observed in the current studies as reported previously [7], and IWR-1 treatment caused a small, but significant reduction in the FSH-induced repression of *CARTPT* mRNA. Results support specificity in potentiation of FSH target gene expression via WNT signaling in bovine granulosa cells.

We also explored the hormonal regulation by FSH of expression of select WNT pathway members and accompanying effects of inhibition of WNT signaling in bovine granulosa cells. Despite increased granulosa cell *DVL1* mRNA expression associated with follicle wave progression, no effect of FSH and or IWR-1 treatment on *DVL-1* expression was observed in the current studies. Interestingly, *FZD6* expression was significantly upregulated by FSH irrespective of IWR-1 treatment (Figure 5B). IWR compounds selectively inhibit the components of canonical WNT pathway that function downstream of LRP and DVL proteins [16]. However, a modest decrease in *FZD6* mRNA was observed in human mesenchymal stem cells subjected to siRNA mediated CTNNB1 knockdown using siRNA [30]. Given granulosa cell expression of *FZD6* was increased coordinate with *CTNNB1* and *DVL1* expression and inversely related to *AXIN2* expression during follicular wave progression, it will interesting to determine in future studies the functional contribution of FZD6 to regulation of FSH action associated with follicle wave progression and dominant follicle selection.

Our results also demonstrated a stimulatory effect of FSH treatment on *AXIN2* and *CTNNB1* mRNA and protein abundance in cultured bovine granulosa cells, but mRNA expression was not impacted by IWR-1 treatment. Previous studies have shown that IWR compound alters the affinity of AXIN2 for CTNNB1 by endo-stabilizing and increasing the level of AXIN2 protein. However, previous studies demonstrated that IWR-1 treatment does not change the *de novo* synthesis or transcription of *AXIN2* and *CTNNB1* [16], [24].

In summary, expression of multiple WNT system components including WNT ligands, FZD receptors and downstream signaling molecules detected in bovine granulosa cells in the current studies support a potential role for canonical and non-canonical WNT pathways during follicular waves in cattle. Observed functional requirement of WNT signaling for multiple components of FSH action detected using a pharmacological approach, coupled with enhanced expression of CTNNB1 and DVL1 and reduced expression of AXIN2 in bovine granulosa cells observed during wave progression to the early dominance stage, supports a potential important role for WNT signaling in potentiating FSH action in the face of declining FSH concentrations characteristic of dominant follicle selection.

## Supporting Information

Table S1 Bovine granulosa cell expressed WNT signaling pathway members identified by RNA sequencing.
(XLSX)
